# Tenofovir-Diphosphate as a Marker of HIV Pre-exposure Prophylaxis Use Among East African Men and Women

**DOI:** 10.3389/fphar.2019.00401

**Published:** 2019-04-17

**Authors:** Maria Pyra, Pete Anderson, Jessica E. Haberer, Renee Heffron, Connie Celum, Stephen Asiimwe, Elly Katabira, Nelly R. Mugo, Elizabeth A. Bukusi, Jared M. Baeten

**Affiliations:** ^1^Department of Epidemiology, University of Washington, Seattle, WA, United States; ^2^Department of Global Health, University of Washington, Seattle, WA, United States; ^3^Department of Pharmaceutical Sciences, University of Colorado, Aurora, Aurora, CO, United States; ^4^Massachusetts General Hospital Global Health, Boston, MA, United States; ^5^Department of Medicine, Harvard Medical School, Boston, MA, United States; ^6^Department of Medicine, University of Washington, Seattle, WA, United States; ^7^Kabwohe Clinical Research Center, Kabwohe, Uganda; ^8^Infectious Diseases Institute, Makerere University, Makerere, Uganda; ^9^Kenya Medical Research Institute, Nairobi, Kenya; ^10^Department of Obstetrics & Gynecology, University of Washington, Seattle, WA, United States

**Keywords:** tenofovir-diphosphate, adherence, pre-exposure prophylaxis, women, Africa, HIV

## Abstract

**Background:** Controlled pharmacokinetic (PK) studies in United States populations have defined categories of tenofovir-diphosphate (TFV-DP) in dried blood spots (DBS) for various pre-exposure prophylaxis (PrEP) adherence targets. It is unknown how these categories perform in other populations. Therefore, we evaluated the sensitivity and specificity of these PK-derived categories compared to daily medication electronic adherence monitoring (MEMS) data among East African men and women using daily PrEP.

**Methods:** Participants were enrolled as members of HIV serodiscordant couples as part of an open-label PrEP study in Kenya and Uganda. Blood samples were taken at quarterly visits and stored as DBS, which were analyzed for TFV-DP concentrations.

**Results:** Among 150 samples from 103 participants, MEMs data indicated that 87 (58%) took ≥4 doses and 62 (41%) took ≥6 per week consistently over the 4 weeks prior to sample collection. Sensitivities of DBS TFV-DP levels were 62% for the ≥4 doses/week category (≥700 fmol/punch TFV-DP) and 44% for the ≥6 doses/week category (≥1050 fmol/punch TFV-DP); specificities were 86 and 94%, respectively. There were no statistically significant differences in these sensitivities and specificities by gender.

**Conclusion:** In this sample of East African PrEP users, categories of TFV-DP concentrations developed from directly observed PrEP use among United States populations had high specificity but lower than expected sensitivity. Sensitivity was lowest when MEMS data indicated high adherence (i.e., ≥6 doses/week). PrEP studies and implementation programs should carefully consider the sensitivity and specificity of the TFV-DP levels used for adherence feedback.

## Introduction

Clinical trials have shown that pre-exposure prophylaxis (PrEP) is highly effective for preventing HIV (Grant et al., 2010; Baeten et al., 2012; Thigpen et al., 2012). However, effectiveness depends strongly on adherence (Abdool Karim, 2014). Clinical studies and open-label implementation programs have used many methods to assess PrEP adherence. As participants may misreport PrEP use, biomarkers are of particular interest as an objective marker of adherence. Some biomarkers, including concentrations of tenofovir in plasma or emtricitabine-triphosphate in blood cells, detect only recent use and are susceptible to white-coat effects, when individuals take a dose before a visit to appear adherent.

In contrast, the active metabolite tenofovir-diphosphate (TFV-DP) accumulates in blood cells in a dose-proportional manner (Anderson et al., 2017) and is a marker of cumulative use over the prior month. As a biomarker, TFV-DP is increasingly being used to assess adherence in research and implementation projects, with adherence counseling sometimes tailored based on these values (Celum and Delaney-Moretlwe, 2015). Intensive, controlled pharmacokinetic studies in United States populations were conducted with directly observed treatment (DOT) to estimate the expected levels of TFV-DP for specific adherence targets (i.e., ≥2 or ≥4 doses/week) (Anderson et al., 2017); these levels have been associated with HIV protection among men who have sex with men in the iPrEx placebo-controlled trial conducted in the United States, South America, Thailand and South Africa (Grant et al., 2014; Liu et al., 2016). However, the sensitivity and specificity of these levels have not specifically been evaluated in African populations, where PrEP roll-out is actively underway, although biological differences are possible (Anderson et al., 2017). Therefore, our goal was to evaluate the sensitivity and specificity of these categories in African men and women, using electronic adherence monitoring data for comparison.

## Materials and Methods

### Study Sample

These data come from the Partners Demonstration Project, an open-label PrEP demonstration study among HIV serodiscordant couples in East Africa, as previously described (Baeten et al., 2016; Haberer et al., 2017). A total of 1,013 couples were enrolled in Kenya and Uganda. Participants were given electronic monitoring devices (MEMS caps, WestRock, Switzerland), which recorded daily bottle openings. MEMS data were downloaded and other variables collected at quarterly study visits, when PrEP (emtricitabine/tenofovir disoproxil fumarate 200/300 mg, prescribed for daily use) was dispensed. Participants received PrEP adherence counseling at study visits, but neither MEMS data nor TFV-DP concentrations were shared. Blood samples were prepared into dried blood spots (DBS) at quarterly visits and stored at -20°C. The University of Washington Human Subjects Division as well as ethics review committees at each site (either the National HIV/AIDS Research Committee of the Uganda National Council for Science and Technology or the Ethics Review Committee of the Kenya Medical Research Institute approved the protocol). All participants provided written informed consent in their preferred language in accordance with the Declaration of Helsinki.

To ensure variation in adherence patterns, we selected 120 random DBS, stratified by gender and evenly distributed with 0–2, 3–5, or 6–7 recorded openings by MEMS in the week prior to collection. In addition, any samples from the same participant at both the first and third month study visit were included (*n* = 76 additional samples), to assess changes over time. To optimize accuracy of MEMS data, we excluded any DBS when the participant reported curiosity openings (opening without removing pills, *n* = 8) or pocket dosing (removing multiple pills, *n* = 17); we also limited the data to one bottle opening per day. Finally, we excluded any visits during pregnancy (*n* = 21) (Pyra et al., 2018). TFV-DP was analyzed from DBS by liquid chromatography tandem mass spectrometry (LC-MS/MS) at the University of Colorado (Castillo-Mancilla et al., 2014, 2016; Zheng et al., 2016); values below the lower limit of quantitation of 31.25 fmol/punch were set to half the lower limit.

### Statistical Analysis

We considered three adherence targets as recorded by MEMS consistently for the 4 weeks prior to sample collection, and corresponding TFV-DP categories based on previous DOT analyses (Castillo-Mancilla et al., 2012; Anderson et al., 2017): ≥700 fmol/punch for ≥4 doses, ≥1050 fmol/punch for ≥6 doses, and ≥1250 fmol/punch for 7 doses/week. The PK thresholds were established at 25th percentiles from prior studies such that 75% of adherent PrEP users were captured by the category.

We reported concentrations of TFV-DP among consistent users by gender and duration of PrEP use (early use, defined as the first month of PrEP, or later use). We tested gender, duration of PrEP use, total doses over the prior month, and study site in a generalized estimating equation model predicting TFV-DP concentration. We reported sensitivity and specificity, using MEMS data as the standard, with Wald 95% confidence intervals. In a sensitivity analysis, we used average (vs. consistent) doses. We also compared sensitivities and specificities by gender, using an interaction term in generalized estimating equations with a binomial distribution to account for repeated observations. In sensitivity analyses, we excluded samples with unquantifiable TFV-DP and repeated the analyses using doses over only the prior week.

We also calculated positive and negative predictive values over a range of adherence levels. These values indicate how likely an individual’s test result correctly predicts adherence and depend not only on sensitivity and specificity but also on the ratio of true positives to false positives, i.e., the prevalence of being adherent. Next, to assess the effect of misclassification, specifically over-reporting, in the MEMS data, we used a multidimensional bias analysis to test a hypothetical scenario where 98% of those truly meeting the adherence targets were captured by MEMS and between 10 and 30% of true low-adherers were misclassified by MEMs as adherent (Lash et al., 2009). Analyses were conducted in SAS 9.4, with bias and predictive value analyses in Excel.

## Results

### Participant Characteristics

Our analysis includes 150 DBS from 103 participants with 55 samples from 35 women and 95 samples from 68 men. The median age of men was 32.9 years and women was 29.5 years at baseline. Men recorded an average of 4.6 doses/week over the prior 4 weeks and women recorded 4.1 doses/week on average (Supplementary Table 1). Twenty-two samples were classified as early PrEP use. Overall, 58% (87) of samples consistently had ≥4 doses, 41% (62) had ≥6 doses, and 14% (21) had 7 doses/week for all of the prior 4 weeks as recorded by MEMS (Supplementary Figure 1).

### Observed Concentrations

The average concentration among samples with ≥4 doses/week by MEMS was 925 fmol/punch [standard deviation (SD) 509] (Table 1). For those with ≥6 doses/week, the average was 994 fmol/punch (SD 517) and for those with 7 doses/week, it was 928 fmol/punch (SD 390). After controlling for site, duration of PrEP use, and total doses by MEMS, the concentration of TFV-DP was 12% higher among women compared to men, which was not statistically significant [adjusted risk difference (aRD) 80.8 fmol/punch (95% CI -78.7, 242.0)]. In the same model, early use (first month of PrEP) was significantly associated with 20% lower concentrations, aRD -129.1 fmol/punch (95% CI -227.8, -30.3).

**Table 1:** TFV-DP concentrations and detection among consistent users, by gender and by use.

	Consistent≥4 weekly doses			Consistent≥6 weekly doses			Consistent 7 weekly doses		
	Women (*n* = 30)	Men (*n* = 57)	Total (*n* = 87)	Women (*n* = 20)	Men (*n* = 42)	Total (*n* = 62)	Women (*n* = 5)	Men (*n* = 16)	Total (*n* = 21)
Mean TFV-DP fmol/punch (SD)	898 (530)	939 (502)	925 (509)	931 (507)	1024 (525)	994 (517)	759 (233)	981 (420)	928 (390)
Unquantifiable TFV-DP, % (n)	7% (2)	4% (2)	5% (4)	5% (1)	2.4% (1)	3% (2)	0% (0)	0% (0)	0% (0)

	**Early use (*n* = 22)**	**Late use (*n* = 65)**	**Total (*n* = 87)**	**Early use (*n* = 18)**	**Late use (*n* = 44)**	**Total (*n* = 62)**	**Early use (*n* = 6)**	**Late use (*n* = 15)**	**Total (*n* = 21)**

Mean TFV-DP fmol/punch (SD)	942 (460)	920 (528)	925 (509)	1011 (447)	988 (548)	994 (517)	1097 (537)	860 (311)	928 (390)
Unquantifiable TFV-DP, % (n)	5% (1)	5% (3)	5% (4)	0% (0)	5% (2)	3% (2)	0% (0)	0% (0)	0% (0)


### Sensitivity and Specificity

The sensitivity of the ≥700 fmol/punch category was 62% for the ≥4 doses/week dosing and the specificity was 86% (Table 2). The sensitivity of the ≥1050 fmol/punch category was lower, 44%, and the specificity was higher, 93%, for ≥6 doses/week dosing. The ≥1250 fmol/punch category had a sensitivity of 19% and a specificity of 90% for the 7 doses/week dosing. There were no statistical differences by gender, though we were unable to test the difference for the ≥1250 category due to small sample size. When excluding 18 samples with unquantifiable TFV-DP, results were similar (Table 2). In addition, results based on doses over the prior 1 week and on average doses were similar to those described (Supplementary Table 2).

**Table 2:** Sensitivity and specificity of TFV-DP categories by consistent doses over prior 4 weeks.

	≥700 fmol/punch (≥4 doses per week)		≥1050 fmol/punch (≥6 doses per week)		≥1250 fmol/punch (7 doses per week)	
	Sensitivity (95% CI)	Specificity (95% CI)	Sensitivity (95% CI)	Specificity (95% CI)	Sensitivity (95% CI)	Specificity (95% CI)
All Samples	62%	86%	44%	93%	19%	90%
	(52%, 72%)	(77%, 94%)	(31%, 56%)	(88%, 98%)	(2%, 36%)	(85%, 95%)
Women	60%	92%	35%	94%	0%	90%
	(42%, 78%)	(81%, 100%)	(14%, 56%)	(87%, 100%)	(na)	(82%, 98%)
Men	63%	82%	48%	92%	25%	90%
	(51%, 76%)	(69%, 94%)	(33%, 63%)	(85%, 100%)	(4%, 46%)	(83%, 97%)
Early use	73%	83%	33%	94%	17%	89%
	(54%, 91%)	(62%, 100%)	(12%, 55%)	(82%, 100%)	(0%, 46%)	(78%, 100%)
Quantifiable TFV-DP	65%	82%	45%	92%	19%	88%
	(55%, 75%)	(71%, 92%)	(32%, 58%)	(85%, 98%)	(2%, 36%)	(82%, 94%)


Using the observed sensitivity and specificity for the ≥700 fmol/punch and ≥1050 fmol/punch categories, we calculated positive and negative predictive values over a range of adherence levels to provide context for interpreting these results. For instance, if more than half of PrEP users are truly meeting the ≥4 doses/week target, there is a >80% probability that a TFV-DP result >700 fmol/punch is identifying a true high- adherent user; however, there is <70% probability that a result <700 fmol/punch is correctly identifying a true low-adherent user (Supplementary Figure 2).

### Misclassification Bias Analysis

We recognized potential for misclassification of adherence from MEMS data. Both over and under-reporting have been observed with electronic monitoring, but over-reporting would explain the low sensitivities we observed. In the bias analysis, we found there would have to be >20% misclassification among true low-adherers for sensitivities to reach the expected level of 75% (Supplementary Table 3).

## Discussion

In this analysis, we assessed the sensitivity and specificity of PK-derived categories from United States studies in TFV-DP concentrations from PrEP-taking populations in Africa. We found that these categories have high specificity but relatively low sensitivities, compared to MEMS data. The sensitivity for the ≥700 fmol/punch category for the ≥4 doses/week [62% (95% CI 52%, 72%)] was closest to the expected 75%. The sensitivity declined to 44% and 19% for ≥ 6 and 7 doses/week, indicating unexpectedly low TFV-DP with high MEMS openings. However, the high specificity for all the categories tested (generally ≥ 85%) means that most low-adherent users would have TFV-DP concentrations below the cut-offs.

In planning to use adherence monitoring, especially for counseling, it is important to consider the relative value of correctly identifying true high-adherent versus true low-adherent users, as sensitivity and specificity are trade-offs. While identifying low-adherent users (i.e., true negatives) may be useful to improve adherence, misidentifying participants who are achieving good adherence (i.e., false negatives) could have undesirable consequences (Van der Straten et al., 2018) including demotivation to take PrEP, although additional work is needed in this area. Studies are needed about how best to frame adherence counseling messages using biomarker feedback.

Our results are in line with the existing pharmacokinetic literature. Previous work has found higher TFV-DP levels in women compared to men with similar patterns of pill-taking (Anderson et al., 2017); we found a similar trend by gender, though it did not reach significance. In addition, TFV-DP is not expected to reach steady state within the first month of use (Anderson et al., 2017); in adjusted analyses, we found significantly lower concentrations from early use compared to later use samples.

The low sensitivities we observed were unexpected; several explanations are possible. TFV-DP concentrations have previously been reported to be approximately 14% lower in African-American participants compared to Caucasians, though this finding was not statistically significant in a small study from the United States (Anderson et al., 2017). Potential mechanisms for this difference are not known, but may include differential expression or function of transporters or enzymes that influence TFV and/or TFV-DP cellular pharmacology [including esterases, P-glycoprotein (ABCB1), breast cancer resistance protein (BCRP), adenylate kinase I, pyruvate kinase, nucleoside diphosphate kinase, or factors influencing red blood cell turnover] (Tong et al., 2007; Laizure et al., 2013; Lade et al., 2015). It may be important to conduct additional controlled PK studies in an African population.

However, over-reporting of adherence by MEMS would also bias the results in the direction we observed. To address this, we conducted a sensitivity bias analysis and found that a large fraction (>20%) of true low-adherent users would have to be misclassified in order to achieve the expected 75% sensitivity. Finally, while sensitivity and specificity are not dependent on prevalence, in this situation, one group taking exactly 4 doses per week and another group taking exactly 7 doses per week would both meet the dichotomized ≥4 doses/week target – but likely have different proportions ≥700 fmol/punch and thereby different sensitivities. However, this would not explain the very low sensitivity for the 7 doses/week target, where there should be no variation.

The major limitation of this analysis, as already discussed, is the use of MEMS data, which may be subject to misclassification. However, MEMS has been shown to be valid of measure of PrEP and ART use (for example, by AUC compared to other biomarkers) and used as the standard in other studies comparing adherence measures (Arnsten et al., 2001; Musinguzi et al., 2016; Abaasa et al., 2017). Finally, we had small sample sizes for some categories that limited analyses.

These results provide important information regarding the sensitivity and specificity of TFV-DP categories in an African population. In addition, even in United States populations, the PK-derived cut-offs were designed to have only 75% sensitivity, which should be taken into account for studies assessing PrEP adherence. Determining categories of TFV-DP as an objective measure of adherence is important for clinical trials evaluating adherence and for counselors and clinicians using drug levels to provide feedback to patients. Indeed, patients have indicated that biomarker feedback is acceptable and even desired (Koester et al., 2015; Van der Straten et al., 2015). When providing biomarker feedback, it is important to consider the desired sensitivity and specificity in different populations, and to design adherence messaging accordingly.

## Members of the Partners Demonstration Project Team

Coordinating Center (University of Washington) and collaborating investigators (Harvard Medical School, Johns Hopkins University, Massachusetts General Hospital): Jared Baeten (protocol chair), CC (protocol co-chair), RH (project director), Deborah Donnell (statistician), Ruanne Barnabas, Jessica Haberer, Harald Haugen, Craig Hendrix, Lara Kidoguchi, Mark Marzinke, Susan Morrison, Jennifer Morton, Norma Ware, Monique Wyatt. Project sites: Kabwohe, Uganda (Kabwohe Clinical Research Centre): SA, Edna Tindimwebwa. Kampala, Uganda (Makerere University): EK, Nulu Bulya. Kisumu, Kenya (Kenya Medical Research Institute): Elizabeth Bukusi, Josephine Odoyo. Thika, Kenya (Kenya Medical Research Institute, University of Washington): Nelly Rwamba Mugo, Kenneth Ngure. Data Management was provided by DF/Net Research, Inc. (Seattle, WA, United States). PrEP medication was donated by Gilead Sciences.

## Ethics Statement

The study was carried out in accordance with the recommendations of the University of Washington Human Subjects Division and ethics review committees at each of the study sites (for Kabwohe and Kampala Uganda, the National HIV/AIDS Research Committee of the Uganda Council for Science and Technology; for Kisumu and Thika, Kenya, the Ethics Review Committee of the Kenya Medical Research Institute). All participants provided written informed consent in their preferred language in accordance with the Declaration of Helsinki.

## Author Contributions

MP and JB designed the research question. PA analyzed the samples. SA, EK, NM, and EB collected the data. MP analyzed the data and drafted the manuscript. All authors contributed to the manuscript.

## Conflict of Interest Statement

The authors declare that the research was conducted in the absence of any commercial or financial relationships that could be construed as a potential conflict of interest.
