# Supplementary material for: Tenofovir-Diphosphate as a Marker of HIV Pre-exposure Prophylaxis Use Among East African Men and Women
Source: Front Pharmacol. 2019 Apr 17;10:401. doi: 10.3389/fphar.2019.00401 (PMC6478885; doi:10.3389/fphar.2019.00401)
Supplement: Supplementary file 1 [file Data_Sheet_1.docx]

**Supplemental Figure 1. TFV-DP Concentrations by Consistent Use Categories & Gender:** TFV-DP levels are presented using three adherence targets according to electronic adherence monitoring (>4 doses, >6 doses, and 7 doses per week, over each of the prior 4 weeks); the reference lines indicate the established PK-derived categories based on 25^th^ percentiles.


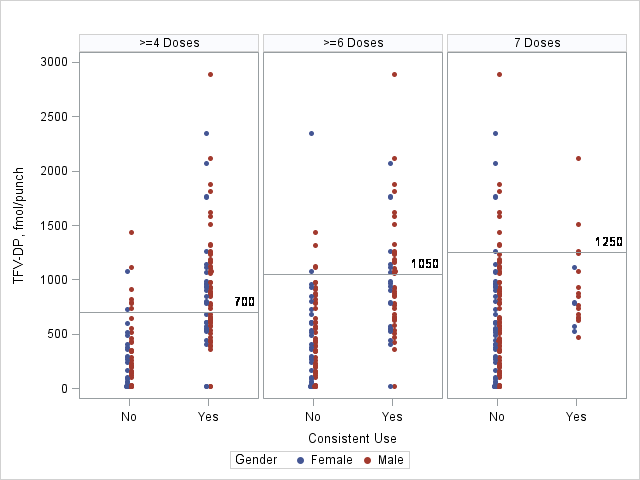


**Supplemental Figure 2. Predictive Values by Adherence in the Population:** The probability that an individual with a TFV-DP result above the cut-off is actually adherent (blue lines) and the probability than an individual with a TFV-DP result below the cut-off is actually non-adherent (orange lines) are presented for two adherence targets (>4 and >6 doses/week) and their associated PK-cutoffs. These probabilities vary according to the proportion of the overall population who are adherent, shown on the x-axis. As an example, the dotted line indicates that if 50% of the population is adherent at >4/week, a positive test means an 80% chance that the individual is adherent, while a negative test means a 70% that the individual is non-adherent.

**
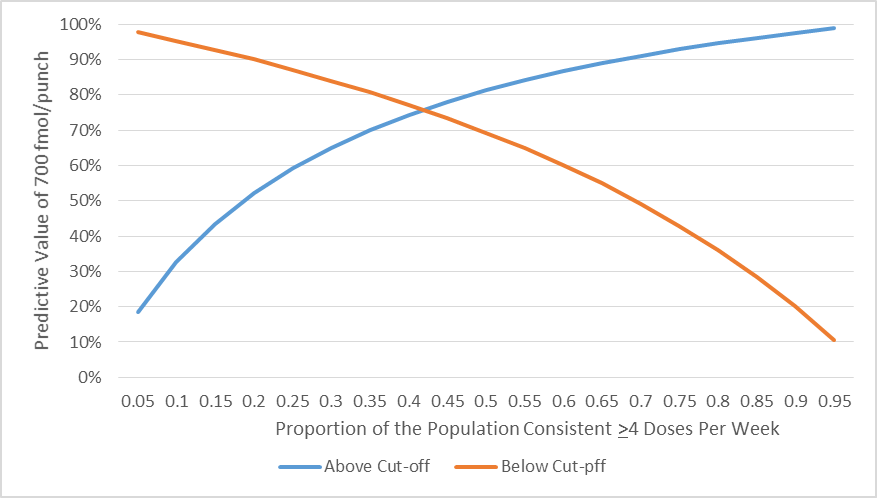

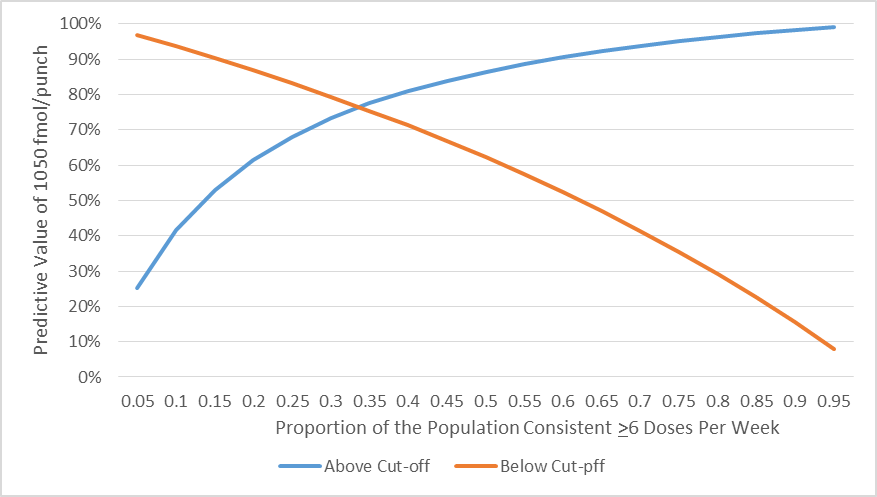
**

**(B)**

**(A)**

**Supplemental Table 1. Characteristics of Samples**

|  | **Women**  **(n=55)** | **Men**  **(n=95)** | **Total**  **(n=150)** |
| --- | --- | --- | --- |
| **Mean Age (SD) at Baseline** | 29.5 (6.0) | 32.9 (10.5) | 31.7 (9.2) |
| **Mean BMI (SD) at Baseline** | 23.9 (4.0) | 22 (2.4) | 23.7 (3.2) |
| **Median creatinine clearance (IQR)** | 100 (87, 114) | 122 (105, 145) | 110.5 (96,136) |
| **Consistent >4 Doses per week by electronic monitoring^*^** | 55% (30) | 60% (57) | 58% (87) |
| **Consistent >6 Doses per week by electronic monitoring^*^** | 36% (20) | 44% (42) | 41% (62) |
| **Consistent 7 Doses per week by electronic monitoring^*^** | 9% (5) | 17% (16) | 14% (21) |
| **Average Doses per Week*(SD)** | 4.1 (2.8) | 4.6 (2.6) | 4.5 (2.6) |
| **Average Doses over Prior Week* (SD)** | 3.6 (2.8) | 4.5 (2.7) | 4.1 (2.7) |
| **^*^ over prior 4 weeks** | | | |

**Supplemental Table 2. Sensitivity & Specificity of TFV-DP Categories by Average Doses over Prior 4 Weeks**

|  | **>700 fmol/punch**  **(>4 Doses per Week)** | | **>1050 fmol/punch**  **(>6 Doses per Week)** | |
| --- | --- | --- | --- | --- |
|  | **Sensitivity**  **(95% CI)** | **Specificity**  **(95% CI)** | **Sensitivity**  **(95% CI)** | **Specificity**  **(95% CI)** |
| **All Samples** | 60%  (50%, 70%) | 89%  (81%, 97%) | 38%  (27%, 50%) | 94%  (88%, 99%) |
| **Women** | 56%  (39%, 73%) | 91%  (80%, 100%) | 27%  (10%, 44%) | 93%  (84%, 100%) |
| **Men** | 62%  (50%, 74%) | 88%  (76%, 99%) | 45%  (30%, 59%) | 94%  (87%, 100%) |

**Supplemental Table 3. Bias Sensitivity Analysis of Misclassification in Electronic Adherence Data**

| **Electronic Adherence Data*** | **Sensitivity of 700 fmol/punch for >4 doses per week** | **Sensitivity of 1050 fmol/punch for >6 doses per week** |
| --- | --- | --- |
| **Observed** | 0.62 | 0.44 |
| **10% Misclassified** | 0.66 | 0.50 |
| **20% Misclassified** | 0.73 | 0.64 |
| **30% Misclassified** | 0.84 | 1.00 |

*Electronic adherence data with a sensitivity of 98% and 90% specificity (10% misclassified as adherent); 80% specificity (20% misclassified as adherent); and 70% specificity (30% misclassified as adherent).
